# Individualised flow-controlled ventilation reduces applied mechanical power and improves ventilation efficiency in a porcine intra-abdominal hypertension model

**DOI:** 10.1186/s40635-024-00608-9

**Published:** 2024-03-07

**Authors:** Julia Abram, Patrick Spraider, Julian Wagner, Gabriel Putzer, Manuela Ranalter, Sarah Rinner, Andrea Katharina Lindner, Bernhard Glodny, Tobias Hell, Tom Barnes, Dietmar Enk, Judith Martini

**Affiliations:** 1 Department of Anesthesiology and Intensive Care Medicine, Medical University Innsbruck, Innsbruck, Austria; 2 Department of Urology, Medical University Innsbruck, Innsbruck, Austria; 3 Department of Radiology, Medical University of Innsbruck, Innsbruck, Austria; 4 Department of Mathematics, Faculty of Mathematics, Computer Science and Physics, University of Innsbruck, Innsbruck, Austria; 5 Professor Emeritus, University of Greenwich, London, UK; 6 Faculty of Medicine, University of Münster, Münster, Germany

**Keywords:** Flow-controlled ventilation, Pressure-controlled ventilation, Intra-abdominal hypertension, Mechanical power, Atelectasis

## Abstract

**Background:**

Aim of this study was to evaluate feasibility and effects of individualised flow-controlled ventilation (FCV), based on compliance guided pressure settings, compared to standard of pressure-controlled ventilation (PCV) in a porcine intra-abdominal hypertension (IAH) model. The primary aim of this study was to investigate oxygenation. Secondary aims were to assess respiratory and metabolic variables and lung tissue aeration.

**Methods:**

Pigs were randomly assigned to FCV (*n* = 9) and PCV (*n* = 9). IAH was induced by insufflation of air into the abdomen to induce IAH grades ranging from 0 to 3. At each IAH grade FCV was undertaken using compliance guided pressure settings, or PCV (*n* = 9) was undertaken with the positive end-expiratory pressure titrated for maximum compliance and the peak pressure set to achieve a tidal volume of 7 ml/kg. Gas exchange, ventilator settings and derived formulas were recorded at two timepoints for each grade of IAH. Lung aeration was assessed by a computed tomography scan at IAH grade 3.

**Results:**

All 18 pigs (median weight 54 kg [IQR 51–67]) completed the observation period of 4 h. Oxygenation was comparable at each IAH grade, but a significantly lower minute volume was required to secure normocapnia in FCV at all IAH grades (7.6 vs. 14.4, MD − 6.8 (95% CI − 8.5 to − 5.2) l/min; *p* < 0.001). There was also a significant reduction of applied mechanical power being most evident at IAH grade 3 (25.9 vs. 57.6, MD − 31.7 (95% CI − 39.7 to − 23.7) J/min; *p* < 0.001). Analysis of Hounsfield unit distribution of the computed tomography scans revealed a significant reduction in non- (5 vs. 8, MD − 3 (95% CI − 6 to 0) %; *p* = 0.032) and poorly-aerated lung tissue (7 vs. 15, MD − 6 (95% CI − 13 to − 3) %, *p* = 0.002) for FCV. Concomitantly, normally-aerated lung tissue was significantly increased (84 vs. 76, MD 8 (95% CI 2 to 15) %; *p* = 0.011).

**Conclusions:**

Individualised FCV showed similar oxygenation but required a significantly lower minute volume for CO_2_-removal, which led to a remarkable reduction of applied mechanical power. Additionally, there was a shift from non- and poorly-aerated lung tissue to normally-aerated lung tissue in FCV compared to PCV.

## Background

Intra-abdominal hypertension (IAH) is a frequent issue in intensive care unit (ICU) patients, with an incidence of one-third at ICU admission and a prevalence of one-half during ICU stay in a mixed ICU population suffering from internal and surgical conditions [1]. In addition to haemodynamic deterioration, IAH can also lead to considerable impairment of renal- and lung function [2, 3] with an overall increase in morbidity and mortality [4]. In particular, lung mechanics is impaired by the transfer of abdominal pressure to the thoracic cavity which is accentuated in the region of the diaphragm. Diaphragmatic excursion is also reduced during ventilation leading to a decrease in both total lung capacity and functional residual capacity, which provoke an increased risk of atelectasis [5, 6]. Positive end-expiratory pressure (PEEP) is commonly increased to counteract the effects of the increased abdominal pressure and reduce the alveolar collapse. However, the optimal PEEP is currently unknown [3]. For example, higher PEEP levels have been shown to increase end-expiratory lung volume but are accompanied by a reduction of cardiac output [6]. IAH has also been shown to be an independent risk factor for ventilator associated pneumonia [7], which further impairs lung mechanics and gas exchange performance.

Flow-controlled ventilation (FCV) is an emerging ventilation mode that could overcome some difficulties of mechanical ventilation and allows accurate titration of PEEP and peak pressure [8]. FCV uses constant gas flow during inspiration and expiration and with direct tracheal pressure measurement, this facilitates precise determination of dynamic compliance. Both PEEP and peak pressure (*P*_peak_) can be accurately set to achieve the highest compliance for each individual patient [8–11]. This strategy has been shown to improve gas exchange parameters [8–10] and substantially reduce applied mechanical power in animal models [8, 10, 12]. This was confirmed in a randomised controlled trial in cardiac surgery patients [13].

In patients suffering from intra-abdominal hypertension, invasive mechanical ventilation frequently exceeds the typical range of lung protective ventilation settings [14, 15]. A ventilation strategy which optimises gas exchange within the limits of lung mechanics and reduces mechanical power may diminish ventilator-induced lung injury in these patients [16–19].

The primary aim of this study was to compare oxygenation (assessed from PaO_2_ measurements) between FCV and standard PCV ventilation. Secondary aims were to compare the minute volume necessary to maintain normocapnia and applied mechanical power between the two ventilation methods. Additionally, lung tissue aeration was assessed at IAH grade 3 by a computed tomography scan.

## Methods

### Study design

This preclinical randomised trial was performed on 12–16 weeks old domestic pigs of both sexes with a body weight of 50–60 kg. The study was conducted at the animal operating facility at the Medical University of Innsbruck between January and April 2021.

### Animal preparation

Animals were fasted overnight with free access to water. Premedication was performed with intramuscular injection of azaperone (4 mg/kg) and atropine (0.01 mg/kg) one hour before transportation to the experimental facility. Deeper sedation was then induced by an intramuscular injection of ketamine (30 mg/kg). An ear vein was then cannulated, and orotracheal intubation performed during spontaneous breathing (endotracheal tube with an internal diameter of 8.0 mm; Willy Rüsch GmbH, Kernen, Germany). Anaesthesia was induced with single boluses of propofol (2 mg/kg) and rocuronium (1 mg/kg) and then maintained through continuous infusion of propofol (6–8 mg/kg/h), remifentanil (0.2–0.3 µg/kg/min) and rocuronium (0.5 mg/kg/h). Normovolaemia was maintained by infusion of balanced crystalloid solution (5–10 ml/kg/h Elomel iso^®^; Fresenius Kabi Austria GmbH, Graz, Austria). In order to prevent septic complications 1.5 g of cefuroxime was administered and repeated after four hours. This regime has been shown to guarantee an appropriate depth of anaesthesia without haemodynamic disturbances [8, 10, 20]. After induction, baseline ventilation was started using volume-controlled ventilation (VCV) (EvitaXL^®^, Dräger Medical, Lübeck, Germany) with a FiO_2_ of 0.3 and a tidal volume (*V*_T_) of 7 ml/kg body weight. PEEP was set to 5 cmH_2_O and the inspiration to expiration ratio (*I*:*E*) to 1:1.5. Breathing frequency was adjusted in order to maintain normocapnia (35–45 mmHg; 4.7–6.0 kPa PaCO_2_).

For invasive arterial pressure monitoring and arterial blood gas sampling an introducer sheath (5 F; Arrow, Reading, PA, USA) was advanced under ultrasound guidance via the femoral artery into the descending aorta. A pulmonary artery catheter (7 F; Edwards Life Science, Irvine, CA, USA) was then placed under radiological guidance into the right pulmonary artery via the right internal jugular vein after ultrasound guided introducer sheath insertion (8.5 F; Arrow, Reading, PA, USA). A pig-tail catheter (8 F; Navarre^®^ Opti-Drain^®^, Bard, Tempe, USA) was placed into the bladder for urine release after ultrasound guided puncture. An esophageal probe (14 F; NutriVent, Sidam S.R.L., Mirandola, MO, Italy) was positioned according to the instruction manual while monitoring the esophageal pressure swing (looking for the highest cardiac oscillations) and performing an occlusion test. Correct placement was then confirmed radiologically.

To induce IAH, a gas-tight tracheostomy tube (Quicktrach II I.D. 4 mm, VBM Medizintechnik GmbH, Sulz a. N., Germany) was placed in the peritoneal cavity. Air was then insufflated and kept at constant pressure using continuous positive airway pressure system (EvitaXL^®^, Dräger, Lübeck, Germany). Additionally, a directional valve allowing flow into the abdominal cavity was inserted in front of the tracheostomy tube to simulate changes of intra-abdominal pressure synchronous with respiration. A right-sided midaxillary mini-thoracotomy was performed after additional local anaesthesia at the level of the 5th to the 6th rib to insert a sleeve for sidestream dark field microscopy (MicroScan, MicroVision Medical, Amsterdam, The Netherlands). At each measurement timepoint, the MicroScan was inserted into the sleeve and an image of the subpleural region was recorded using a short (1–2 s) inspiratory hold.

### Experimental protocol

After animal preparation baseline measurements were obtained during VCV ventilation. Subsequently the animals were randomly allocated to FCV or PCV and after 1 h without IAH, IAH at grade 1 (15 mmHg = 20 cmH_2_O), followed by grade 2 (20 mmHg = 26 cmH_2_O) and then grade 3 (25 mmHg = 33 cmH_2_O) was induced. The IAH grade was held at each value for one hour. In the FCV arm of the experiment, FCV (Evone^®^, Ventinova Medical B.V., Eindhoven, the Netherlands) was performed with compliance guided PEEP and peak pressure settings [8]. The flow was adjusted to maintain normocapnia and the *I*:*E* ratio was set to 1:1 to minimise overall gas flow and applied mechanical power. In the PCV arm, PCV (EvitaXL^®^, Dräger, Lübeck, Germany) was performed with compliance guided PEEP titration and the peak pressure set to achieve a tidal volume of 7 ml/kg. The respiratory rate was adjusted to maintain normocapnia at an *I*:*E* ratio of 1:1.5 in order to avoid air trapping during uncontrolled expiration. At the end of the protocol computed tomography images were taken of the chest at inspiratory hold. Measurement time points were defined as baseline (T0) and timepoints T1–T8 every 30 min after start of FCV or PCV until the observation period ended after 4 h.

### Respiratory and cardiovascular measurements

Respiratory and cardiovascular measurements were taken at each time point (T0–T8) as defined above. PEEP and *P*_peak_ pressure were directly recorded in both PCV and FCV and esophageal pressure (*P*_es_) was measured at the end of expiration. In the PCV animals a regular check for intrinsic PEEP was performed to rule out air trapping (using the expiratory hold manoeuvre automatically performed by the EvitaXL). Respiratory rate (RR), minute volume (MV) and *V*_T_ were directly recorded from the ventilator. Applied mechanical power was calculated according to published surrogate formulas [21]. Arterial blood gas samples were obtained and pH, arterial partial pressure of CO_2_ (PaCO_2_) and O_2_ (PaO_2_) were measured (ABL800 Flex^®^; Radiometer, Brønshøj, Denmark).

Cardiovascular monitoring included heart rate (HR), mean arterial pressure (MAP) and mean pulmonary artery pressure (MPAP). Cardiac output (CO), systemic and pulmonary vascular resistance (SVR, PVR) were measured via the pulmonary artery catheter after three consecutive injections of 10 ml of saline. Indexes for CO, SVR and PVR were calculated using the predicted body surface area for pigs [22].

### Computed tomography

To assess inspiratory lung aeration a CT scan was performed with an appropriate hold manoeuvre lasting ~ 5 s to obtain an image of the lung at the end of the study protocol at IAH grade 3. The ventilation settings remained otherwise unchanged. All examinations were done with a Somatom Confidence^®^ CT scanner (Siemens Healthineers, Erlangen, Germany). The settings were as follows: tube voltage 120 kV, tube current 600 mA (without exposure modulation), single collimation width 0.6 mm, slice thickness 0.75 mm, total collimation width 19.2 mm, table speed 57.6 mm, table feed per rotation 28.8 mm, spiral pitch 1.5, matrix 512 × 512, window center 50/− 600, window width 350/1200 Hounsfield unit, convolution kernel I40f/3 and B70F, and a field of view 294 mm. For image processing an AW Server Workstation (AWS Version 3.2, Volume Viewer program; General Electric, Boston, MA, USA) was used. The lungs were segmented semi-automatically and the total lung volume then determined automatically, as well as the lung volumes at different Hounsfield unit (HU) thresholds in 20 HU intervals. Non-aerated lung tissue was defined as absorption values between 100 and − 100 HU, poorly aerated lung tissue as values between − 101 and − 500 HU, normally aerated lung tissue as values between − 501 and − 900 HU, and airway as well as overinflated lung tissue as values between − 901 HU and − 1000 HU [23].

### Statistical analysis

The sample size calculation was based on the results of a preclinical ARDS study conducted by our study group [10], which examined oxygenation as a primary outcome parameter. Assuming a similar effect size of 1.5 in this extrapulmonary ARDS model and using a two-sided significance level of 5% and a power of 0.8, we concluded that a sample size of 9 animals per group was appropriate. A mathematician (TH) not involved in the study procedures performed the statistical analyses using R, version 4.0.3 (The R Foundation, Vienna, Austria). For the characteristics of laboratory animals before the start of the experiment, continuous data are presented as median (25th to 75th percentile) and categorical variables as frequencies (%). Effect size and precision are shown with estimated median differences between groups for continuous data and odds ratios for binary variables with 95% confidence intervals (CI). The Wilcoxon rank sum test and Fisher's exact test were applied to assess differences between the groups.

The course of haemodynamic parameters during the entire observation period are shown per group using the median course with corresponding 95% CI’s. Differences between groups were assessed using linear mixed-effects models with random intercepts for time points and subjects as well as the group as fixed effect. Effects in each group by different IAH grade were also analysed with linear mixed-effects model.

All statistical assessments were two-sided, and a significance level of 5% was used.

## Results

Eighteen pigs (FCV *n* = 9; PCV *n* = 9) were enrolled into the study and all animals (median weight 54 kg [IQR 51–67]) completed the 4 h study. Baseline characteristics were comparable between groups except PaO_2_, which was slightly higher in the FCV group (Table 1).

**Table 1:** Demographic data and baseline characteristics of animals

	Total^a^ (*n* = 18)	FCV^a^ (*n* = 9)	PCV^a^ (*n* = 9)	Estimate with 95% CI^b^	*p*-value^c^
Demographic data					
Weight	54 (51–67)	52 (49–57)	56 (51–70)	− 5 (− 21 to 2)	0.1840
Sex [female]	5/18 (27.8%)	3/9 (33.3%)	2/9 (22.2%)		1
Haemodynamic parameters					
HR [/min]	70 (66–76)	67 (65–74)	72 (69–76)	− 5 (− 18 to 4)	0.1995
MAP [mmHg]	79 (74–84)	81 (75–85)	77 (71–81)	4 (− 4 to 12)	0.2689
CVP [mmHg]	13 (11–16)	14 (11–16)	12 (12–13)	1 (− 4 to 4)	0.8239
MPAP [mmHg]	26 (24–28)	27 (25–28)	25 (24–27)	1 (− 5 to 4)	0.6258
PCWP [mmHg]	15 (13–17)	15 (14–17)	14 (13–16)	1 (− 4 to 3)	0.7216
CI [l/min/m^2^]	5.8 (4.8–6.8)	5.6 (4.7–6.9)	5.9 (5.3–6.5)	− 0.6 (− 1.9 to 1.0)	0.4363
PVRI [dyn·s/cm^5^/m^2^]	130 (110–177)	145 (113–208)	125 (108–151)	26 (− 23 to 89)	0.3865
SVRI [dyn·s/cm^5^/m^2^]	847 (670–1031)	1014 (841–1050)	734 (624–875)	228 (− 23 to 533)	0.0503
Respiratory parameters					
RR [/min]	35 (32–39)	34 (32–39)	36 (32–38)	0 (− 6 to 6)	0.9292
*V*_T_ [ml/kg]	7.0 (7.0–7.1)	7.0 (7.0–7.1)	7.0 (7.0–7.1)	0.0 (− 0.1 to 0.1)	0.8945
MV [l/min]	15.1 (12.8–16.0)	14.2 (11.4–15.8)	15.5 (14.1–16.0)	− 1.6 (− 4.7 to 1.0)	0.3770
*P*_peak_ [cmH_2_O]	22 (21–25)	22 (21–23)	22 (21–28)	− 2 (− 7 to 2)	0.4227
PEEP [cmH_2_O]	5	5	5		
ΔP [cmH_2_O]	17 (16–20)	17 (16–18)	17 (16–23)	− 2 (− 7 to 2)	0.4227
C [ml/cmH_2_O]	36.6 (32.6–43.1)	37.0 (33.2–39.6)	35.8 (32.4–49.5)	− 1.4 (− 12.4 to 6.3)	0.8252
C_Calc_ [ml/cmH_2_O]	23.2 (20.8–24.3)	23.1 (20.6–25.6)	23.3 (21.3–24.0)	0 (− 3.8 to 4.6)	0.9647
R [cmH_2_O·s/l]	6.8 (6.6–7.3)	6.8 (6.7–7)	6.8 (6.6–7.4)	0.0 (− 1.7 to 0.6)	1
MP [J/min]	22.7 (18.1–26.8)	22.3 (16.6–24.6)	24.3 (21.0–31.3)	− 4.9 (− 13.4 to 1.8)	0.2224
PaCO_2_ [mmHg]	43 (42–45)	43 (41–45)	43 (42–45)	0 (− 3 to 3)	1
PaO_2_ [mmHg]	116 (112–125)	125 (123–132)	114 (105–115)	15 (6 to 22)	0.017*
Metabolic parameters					
pH	7.38 (7.36–7.40)	7.37 (7.34–7.40)	7.39 (7.38–7.40)	− 0.02 (− 0.05 to 0.02)	0.4265
Hb [mg/dl]	8.8 (8.5–9.12)	8.9 (8.5–9.3)	8.6 (8.5–8.9)	0.2 (− 0.5 to 0.8)	0.6243
Lac [mg/dl]	12 (8.5–16)	12 (10–13)	12 (7–17)	0 (− 5 to 5)	1
SvO_2_ [%]	62 (56–63)	60 (56–63)	63 (57–63)	− 2 (− 7 to 3)	0.5457

*C* displayed compliance from the ventilator, *C*_*Calc*_ calculated static compliance, *CI* cardiac index, *CVP* central venous pressure, *FCV* flow-controlled ventilation, *Hb* arterial hemoglobin level, *HR *heart rate, *Lac* arterial lactate level, *MAP* mean arterial pressure, *MP* mechanical power, *MPAP *mean pulmonary arterial pressure, *MV* respiratory minute volume, *PaCO*_*2*_ arterial partial pressure of carbon dioxide, *PaO*_*2*_ arterial partial pressure of oxygen, *PCV *pressure-controlled ventilation, *PCWP* pulmonary capillary wedge pressure, *PEEP* positive end-expiratory pressure, *pH* arterial potential of hydrogen, *P*_*peak*_ peak pressure, *PVRI *pulmonary vascular resistance index, *R* resistance, *RR* respiratory rate, *SvO*_*2*_ mixed venous oxygen saturation, *SVRI* systemic vascular resistance index, *V*_*T*_ tidal volume, *ΔP *driving pressure (= peak pressure – positive end-expiratory pressure)

^a^Binary data are presented as no./total no. (%), continuous data as medians (25th to 75th percentile)

^b^Odds ratios for binary variables and estimated median difference for continuous variables

^c^Assessed by Fisher’s exact test for categorical variables and Wilcoxon rank sum test for continuous variables, significant differences (*p* < 0.05) are marked with an asterisk

**Fig. 1:**
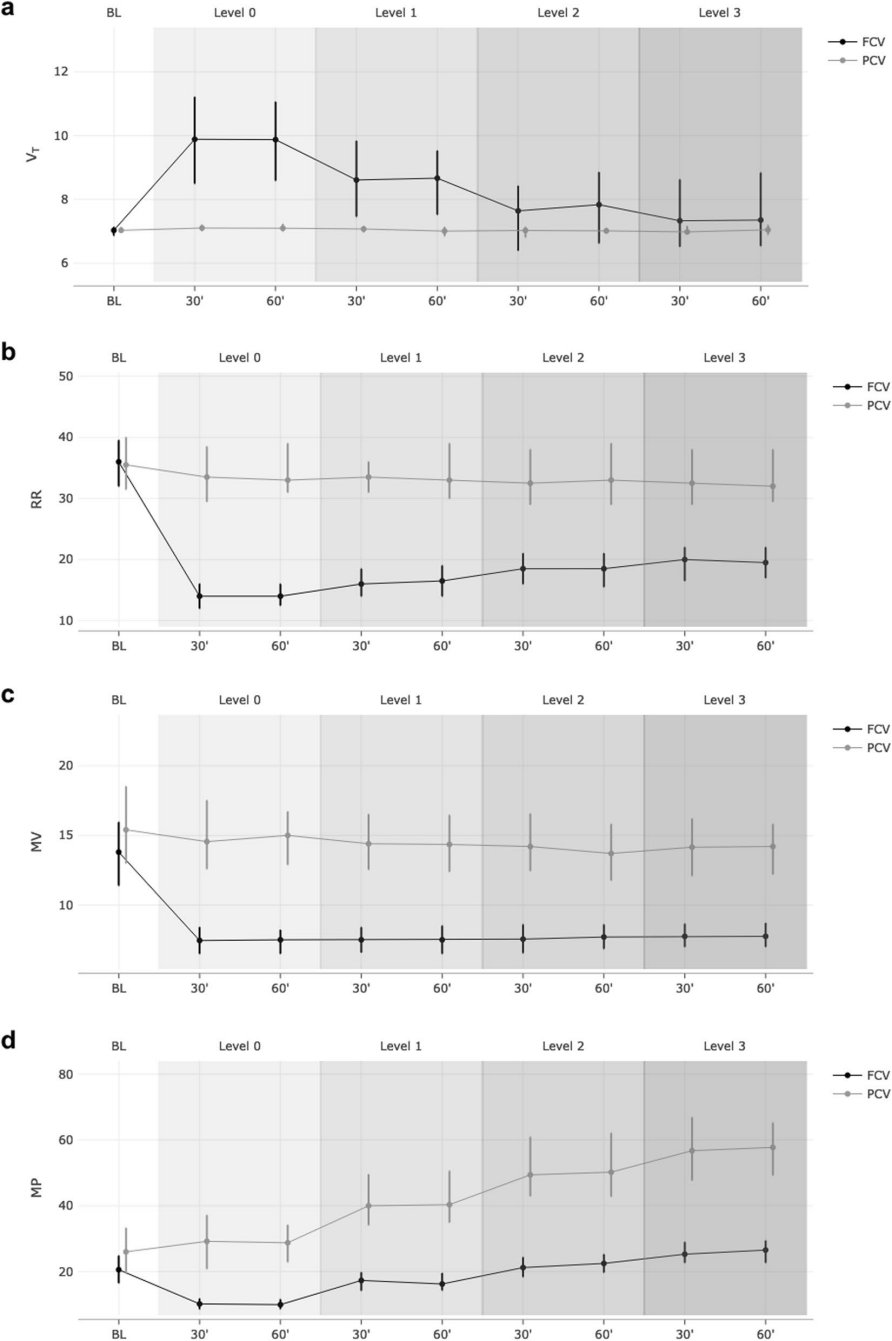
Course of respiratory parameters at baseline (BL) and after randomisation to FCV (black) or PCV (grey) at intra-abdominal hypertension (IAH) grade 0–3. **a** Tidal volume (V_T_ [ml/kg]). **b** Respiratory rate (RR [/min]). **c** Respiratory minute volume (MV [l/min]). **d** Mechanical power (MP [J/min])

The primary outcome measure, PaO_2_, was comparable between groups during the entire course of the protocol (116 vs. 107, MD 9 (− 1 to 19) mmHg; *p* = 0.111) and at each IAH grade (Table 2). The secondary outcome parameter of required MV to maintain normocapnia was substantially lower in FCV (7.6 vs. 14.4, MD − 6.8 (− 8.5 to − 5.2) l/min; *p* < 0.001) during the intervention period, and this finding was similar at each IAH level (Fig. 1). Concomitantly, the calculated applied mechanical power was also substantially lower in FCV compared to PCV animals (18.7 vs. 44.8, MD − 26.1 (− 32.5 to − 19.7) J/min; *p* < 0.001) and increased with increases in IAH level in both groups (Fig. 1).

**Table 2:** Course of respiratory parameters during intra-abdominal hypertension with estimated differences between groups

IAH	FCV^a^ (*n* = 9)	PCV^a^ (*n* = 9)	Estimate with 95% CI^b^	*p*-value^c^
*V*_T_ [ml/kg]				
Grade 0	9.8	7.1	2.7 (1.7 to 3.7)	< 0.001*
Grade 1	8.6	7.0	1.5 (0.6 to 2.4)	0.0039*
Grade 2	7.7	7.0	0.7 (− 0.1 to 1.4)	0.0941
Grade 3	7.6	7.0	0.5 (− 0.4 to 1.4)	0.2606
RR [/min]				
Grade 0	14	34	− 20 (− 23 to − 16)	< 0.001*
Grade 1	16	34	− 18 (− 21 to − 14)	< 0.001*
Grade 2	18	34	− 15 (− 19 to 11)	< 0.001*
Grade 3	19	33	− 14 (− 18 to − 10)	< 0.001*
MV [l/min]				
Grade 0	7.5	14.8	− 7.4 (− 9.1 to − 5.6)	< 0.001*
Grade 1	7.4	14.5	− 7.0 (− 8.8 to − 5.3)	< 0.001*
Grade 2	7.6	14.1	− 6.6 (− 8.2 to − 4.9)	< 0.001*
Grade 3	7.8	14.2	− 6.4 (− 8.0 to − 4.8)	< 0.001*
MP [l/min]				
Grade 0	10.1	28.9	− 18.8 (− 23.8 to − 13.8)	< 0.001*
Grade 1	17.0	41.7	− 24.7 (− 31.4 to − 18.0)	< 0.001*
Grade 2	22.0	51.2	− 29.2 (− 37.2 to − 21.3)	< 0.001*
Grade 3	25.9	57.6	− 31.7 (− 39.1 to − 24.3)	< 0.001*
PEEP [mmHg]				
Grade 0	3	5	− 2 (− 2 to − 2)	0.9804
Grade 1	10	9	1 (0 to 2)	0.9539
Grade 2	15	14	2 (− 1 to 4)	0.1890
Grade 3	18	16	2 (− 1 to 4)	0.1924
*P*_peak_ [mmHg]				
Grade 0	21	20	1 (− 1 to 3)	0.1935
Grade 1	33	29	4 (1 to 6)	0.0141*
Grade 2	40	37	4 (1 to 6)	0.0045*
Grade 3	46	41	5 (2 to 7)	0.0034*
*P*_es_ [cmH_2_O]				
Grade 0	9.9	10.7	− 0.7 (− 3.2 to 1.7)	0.5609
Grade 1	15.4	14.9	0.5 (− 3.8 to 4.7)	0.8395
Grade 2	19.5	17.8	1.7 (− 3.6 to 7.0)	0.5417
Grade 3	22.9	19.6	3.4 (− 2.4 to 9.1)	0.2654
*C* [ml/cmH_2_O]				
Grade 0	27.2	38.7	− 11.5 (− 18.8 to − 4.2)	0.0072*
Grade 1	19.4	25.1	− 5.6 (− 9.7 to − 1.5)	0.0160*
Grade 2	15.9	20.2	− 4.3 (− 7.6 to − 1.0)	0.0214*
Grade 3	13.9	18.2	− 4.2 (− 7.7 to − 0.7)	0.0304*
*C*_calc_ [ml/cmH_2_O]				
Grade 0	35.5	29.3	6.2 (1.0 to 11.3)	0.0325*
Grade 1	24.9	22.4	2.5 (− 0.9 to 5.9)	0.1728
Grade 2	20.9	18.7	2.3 (− 1.7 to 6.2)	0.2719
Grade 3	18.9	16.9	2.0 (− 3.8 to 7.8)	0.5128
*R* [cmH_2_O·s/l]				
Grade 0	6.3	7.2	− 0.9 (− 2.0 to 0.2)	0.1170
Grade 1	7.6	8.1	− 0.5 (− 1.4 to 0.5)	0.3396
Grade 2	9.0	8.9	0.2 (− 0.9 to 1.2)	0.7726
Grade 3	9.6	9.6	0.0 (− 1.6 to 1.7)	0.9790
PaO_2_ [mmHg]				
Grade 0	118	114	4 (− 6 to 14)	0.4022
Grade 1	114	107	7 (− 2 to 16)	0.1301
Grade 2	114	102	12 (− 1 to 25)	0.0918
Grade 3	116	104	12 (− 1 to 25)	0.0864
PaCO_2_ [mmHg]				
Grade 0	42	42	0 (− 2 to 2)	0.8983
Grade 1	43	42	1 (− 2 to 4)	0.5619
Grade 2	43	41	2 (0 to 4)	0.0999
Grade 3	43	42	2 (− 1 to 4)	0.1916

*C* displayed compliance from the ventilator, *C*_*Calc*_ calculated static compliance, *FCV *flow-controlled ventilation, *IAH* intra-abdominal hypertension, *MP* mechanical power, *MV* respiratory minute volume, *PaCO*_*2*_ arterial partial pressure of carbon dioxide, *PaO*_*2*_ arterial partial pressure of oxygen, *PCV* pressure-controlled ventilation, *PEEP* positive end-expiratory pressure, *P*_*es*_ end-expiratory esophageal pressure, *P*_*peak*_ peak pressure, *R* resistance, *RR* respiratory rate, *V*_*T*_ tidal volume, *ΔP* driving pressure (= peak pressure – positive end-expiratory pressure)

^a^Continuous data presented as medians

^b^Estimated median difference for continuous variables

^c^Assessed by Wilcoxon rank sum test for continuous variables, significant differences (*p* < 0.05) are marked with an asterisk

Compliance guided setting of both PEEP and *P*_peak_ was used in FCV but in PCV only the PEEP was set according to the measured compliance and *P*_peak_ was adjusted to achieve a fixed tidal volume of 7 ml/kg. The PEEP level was therefore comparable between groups. However, *P*_peak_ was similar without any IAH but became significantly higher in FCV as the IAH grade increased (Table 2). We estimated the static compliance in FCV using the methods described in [24, 25] to correct for airway resistance and then compared with the static compliance obtained during PCV. We found higher values after compliance guided pressure settings in FCV without any IAH, but similar values at higher IAH grades (Table 2).

Haemodynamic parameters did not differ between FCV and PCV (Table 3).

**Table 3:** Course of haemodynamic parameters during intra-abdominal hypertension with estimated differences between groups

IAH	FCV^a^ (*n* = 9)	PCV^a^ (*n* = 9)	Estimate with 95% CI^b^	*p*-value^c^
HR [/min]				
Grade 0	65	69	− 4 (− 10 to 2)	0.1771
Grade 1	64	65	− 1 (− 8 to 6)	0.7495
Grade 2	64	62	2 (− 6 to 9)	0.6609
Grade 3	65	60	5 (− 6 to 16)	0.4307
MAP [mmHg]				
Grade 0	76	74	1 (− 5 to 7)	0.6916
Grade 1	82	83	− 1 (− 7 to 5)	0.7650
Grade 2	87	87	0 (− 9 to 9)	1
Grade 3	93	90	3 (− 6 to 12)	0.5040
MPAP [mmHg]				
Grade 0	25	26	− 1 (− 4 to 3)	0.7586
Grade 1	32	34	− 2 (− 7 to 3)	0.5173
Grade 2	35	35	1 (− 4 to 5)	0.7806
Grade 3	40	36	3 (− 1 to 7)	0.1224
CVP [mmHg]				
Grade 0	13	14	− 1 (− 4 to 3)	0.7658
Grade 1	20	21	0 (− 4 to 3)	0.8168
Grade 2	25	23	2 (− 1 to 5)	0.2497
Grade 3	27	24	3 (0 to 7)	0.0809
PCWP [mmHg]				
Grade 0	14	15	− 1 (− 4 to 2)	0.4726
Grade 1	20	21	− 1 (− 5 to 3)	0.6657
Grade 2	21	21	0 (− 3 to 4)	0.8261
Grade 3	23	22	1 (− 3 to 4)	0.7519
CI [l/min/m^2^]				
Grade 0	5.3	5.7	− 0.4 (− 1.3 to 0.5)	0.3783
Grade 1	4.8	5.6	− 0.7 (− 1.4 to − 0.1)	0.0407*
Grade 2	4.6	4.9	− 0.3 (− 1.0 to 0.3)	0.3469
Grade 3	3.8	4.2	− 0.5 (− 1.0 to 0.0)	0.0661
SVRI [dyn·s/cm^5^/m^2^]				
Grade 0	990	788	202 (3 to 401)	0.0641
Grade 1	1024	798	226 (31 to 421)	0.0373*
Grade 2	1106	965	141 (− 143 to 426)	0.3455
Grade 3	1427	1098	329 (26 to 632)	0.0502
PVRI [dyn·s/cm^5^/m^2^]				
Grade 0	170	148	23 (− 24 to 69)	0.3529
Grade 1	205	155	50 (0 to 100)	0.0656
Grade 2	250	206	45 (− 44 to 133)	0.3383
Grade 3	346	237	110 (16 to 204)	0.0362*

*CI* cardiac index, *CVP* central venous pressure, *FCV* flow-controlled ventilation, *HR* heart rate, *MAP* mean arterial pressure, *MPAP* mean pulmonary arterial pressure, *PCV *pressure-controlled ventilation, *PCWP* pulmonary capillary wedge pressure, *PVRI* pulmonary vascular resistance index, *SVRI* systemic vascular resistance index

^a^Continuous data are presented as medians

^b^Estimated median difference for continuous variables

^c^Assessed by Wilcoxon rank sum test for continuous variables, significant differences (*p* < 0.05) are marked with an asterisk

**Fig. 2:**
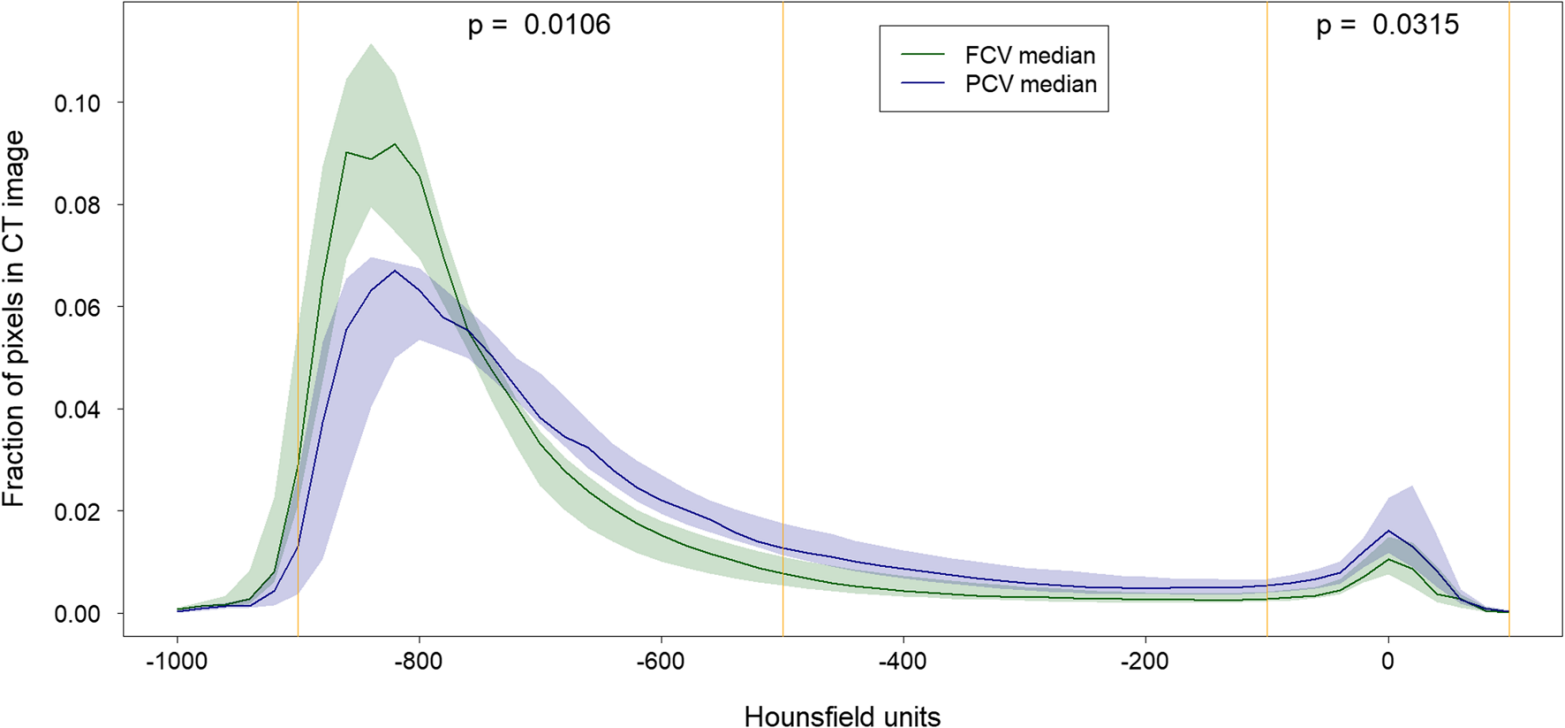
Hounsfield unit (HU) distribution at intra-abdominal hypertension grade 3. Lung tissue aeration defined as non-aerated (HU 100 to − 100), poorly-aerated (HU − 101 to − 500), normally-aerated (HU − 501 to − 900), and overinflated (HU − 901 to − 1000) revealed significant differences in FCV (green line) compared to PCV (blue line). The amount of non- and poorly-aerated lung tissue was lower in FCV, normally-aerated and overinflated lung tissue were more frequent, indicating improved aeration with FCV

Analysis of the Hounsfield unit distributed in the computed tomography scans revealed a significant shift from non-aerated (5 vs. 8, MD − 3 (95% CI − 6 to 0) %; *p* = 0.032) and poorly-aerated (7 vs. 15, MD − 6 (95% CI − 13 to − 3) %; *p* = 0.002) lung tissue to normally-aerated (84 vs. 76, MD 8 (95% CI 2 to 15) %; *p* = 0.011) and overinflated (1 vs. 1, MD 1 (95% CI 0 to 2) %; *p* = 0.040) lung tissue (Table 4; Fig. 2) in FCV.

**Table 4:** Lung aeration assessed with Hounsfield unit distribution from computed tomography scans at IAH grade III

lung tissue	Total^a^ (*n* = 18)	FCV^a^ (*n* = 9)	PCV^a^ (*n* = 9)	Estimate with 95% CI^b^	*p*-value^c^
Overinflated [%]	1 (1–2)	1 (1–3)	1 (0–1)	1 (0 to 2)	0.0400*
Normally-aerated [%]	81 (75–84)	84 (81–88)	76 (72–80)	8 (2 to 15)	0.0106*
Poorly-aerated [%]	11 (8–15)	7 (7–9)	15 (11–21)	− 6 (− 13 to − 3)	0.0019*
Non-aerated [%]	6 (4–8)	5 (4–6)	8 (7–10)	− 3 (− 6 to 0)	0.0315*

*FCV* flow-controlled ventilation, *IAH* intra-abdominal hypertension, *PCV* pressure-controlled ventilation

^a^Continuous data are presented as medians (25th to 75th percentile)

^b^Estimated median difference for continuous variables

^c^Assessed by Wilcoxon rank sum test for continuous variables, significant differences (*p* < 0.05) are marked with an asterisk

## Discussion

In this experimental model of intra-abdominal hypertension, FCV with individually optimised settings resulted in similar oxygenation compared to standard of care PCV. However, the MV required to maintain normocapnia was reduced by 48% in FCV compared to PCV. This led to a reduction of applied mechanical power by 58%. In addition, the CT analyses showed improved lung aeration in FCV.

The primary outcome measure PaO_2_ did not differ between groups, which is an intriguing finding at first sight and is in contrast to previous studies in which individualised FCV resulted in improved oxygenation compared to PCV [8–10, 13]. In addition, the reduction of non-aerated and poorly-aerated lung tissue found in this study would be expected to be associated with improved oxygenation. However, the PaO_2_/FiO_2_ ratio remained well above 300 in all animals and increasing the IAH grade from 1 to 3 did not lead to respiratory failure (according to the Berlin criterion [26]) in any group. It is worth noting that no deterioration of arterial oxygen tension was found in a previous porcine IAH model [6], where PEEP level was matched to IAH grade in the intervention group. Rather, in that study, only an increase in end-expiratory lung volume was observed. It is then possible that, in the early phase of IAH as simulated in our study, oxygenation may not be the primary issue. This might only become apparent later, when ventilator-induced lung injury leads to extrapulmonary acute respiratory distress syndrome. However, this is a hypothesis only and needs to be investigated in further studies.

**Fig. 3:**
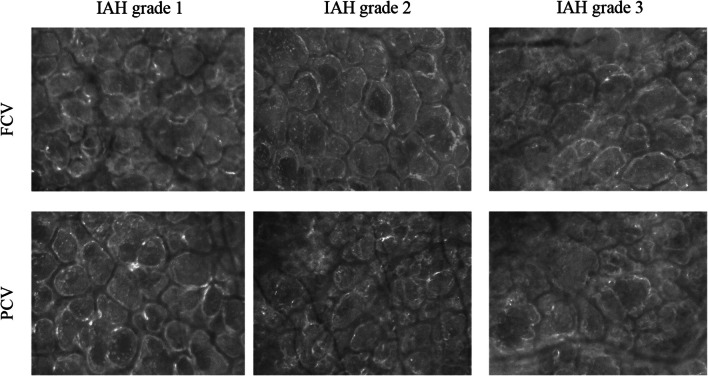
Representative sidestream dark field microscopy images of subpleural lung tissue. Whereas during slighly increased abdominal pressure at IAH grade 1 alveolar size seems to be comparable, in the pictures of the animals ventilated with FCV, a more even ventilation of the individual alveoli directly adjacent to each other becomes apparent at IAH grade 2 and 3

Since the amount of carbon dioxide produced must be comparable for the animals in both groups, the effectiveness of CO_2_ removal can be measured by the minute volume necessary to achieve normocapnia in the blood. The reduction of required MV for similar CO_2_ elimination that we observed in FCV demonstrated improved ventilation efficiency in FCV compared to PCV. Several factors contribute to this: as observed in previous trials [8, 9], under normal circumstances in lung healthy individuals using compliance guided PEEP and *P*_peak_ setting in FCV will increase the tidal volume within the limits of lung mechanics. This reduces the dead space ventilation fraction whilst increasing that for alveolar ventilation. This in turn increases ventilation effectiveness by removing more CO_2_ per breath. However, when this approach is used on injured lungs, as it is the case in a scenario of IAH, lung mechanics are substantially altered (for the worse) and so the compliance guided setting process will result in smaller tidal volume as lung function deteriorates [10, 11]. It is therefore plausible that the tidal volume using compliance guided pressure titration in FCV will decrease as IAH increases. We observed this during this study (Fig. 1). The difference in tidal volumes between FCV and PCV (where it was kept at 7 ml/kg) reduced at increased IAH levels—and indeed was no longer significant at IAH grades 2 and 3. Notwithstanding this, at the comparable tidal volumes using in FCV and PCV during IAH grade 3 the required minute volume to obtain normocapnia was still substantially lower in FCV. It then appears that the dead-space ventilation fraction alone does not provide a satisfactory explanation for the improved ventilation efficiency in FCV compared to PCV (especially at the higher IAH grades). Better CO_2_ removal in FCV was also observed in previous studies where identical tidal volumes were used [27–29] (and therefore a comparable dead space ventilation fraction). We estimated the actual dead space in the experiment, using an apparatus dead space of 62 ml in FCV and 75 ml in PCV (values determined from volumetric measurements of the apparatus) with an assumed anatomical dead space of 2 ml/kg. We could then calculate the alveolar minute ventilation which was of 5.2 l/min in FCV versus 8.4 l/min in PCV without IAH and 4.6 l/min in FCV versus 8.0 l/min in PCV during IAH grade 3. The apparently more efficient CO_2_ removal might be explained if we note that the slow and constant gas flow used in FCV might allow more effective equilibration of lung compartments with different time constants throughout the ventilation cycle, resulting in overall increased CO_2_ removal from the alveolar space [30]. Indeed, the CT analysis we did showed a more even gas distribution in animals ventilated with FCV compared to those in which PCV was used, which is suggestive of a less pronounced preferential gas distribution to faster lung compartments in the FCV animals. This is in line with CT analyses and electrical impedance tomography images from previous trials comparing FCV to VCV and PCV [8, 27, 31]. The hypothesis of a more homogeneous aeration of the entire lung with FCV is also supported by our observation of a more balanced aeration of adjacent alveoli using side-stream dark field microscopy (Fig. 3).

The concept of individualised FCV was developed to minimise physical energy applied to and dissipated in the lung tissue during mechanical ventilation [32, 33]. In this study individualised FCV was accompanied by a reduction of applied mechanical power by 58% compared to PCV. Previous preclinical trials [8, 10, 12] have also demonstrated a significant reduction in mechanical power during FCV compared to PCV or VCV. In this study we found that the ratio of overall elastance to chest wall elastance did not change as IAH increased (the ratio of *P*_peak_ to *P*_es_ was stable at 0.5 during all timepoints of both groups, Table 2) [34]. This was surprising, as an increase in chest wall elastance would be expected [35]. The effect could possibly be due to our porcine model, in which the elasticity is already high and may then increase to a greater extent. However, based on our data we think that the increased mechanical power probably affects both the chest wall, and the lung tissue in our IAH model.

There is increasing evidence that mechanical power might be an appropriate predictor of pulmonary complications [35–37] and mortality in ICU populations both with [38–40] and without acute respiratory distress syndrome (ARDS) [19], although there is currently no clinical implication for this concept. Individually optimised FCV may be a promising strategy to minimise applied mechanical power in the clinic because compliance guided PEEP and *P*_peak_ settings for each individual during FCV maximise compliance and optimise tidal volume, driving pressure, respiratory rate and gas flow—all of which are variables which affect mechanical power [16]. It is also worth noting that mechanical power as usually calculated uses only the energy applied by the ventilator during inspiration, rather than the energy dissipated in the lung tissue over a complete ventilation cycle. While these two quantities are probably related to each other it is probably the energy absorbed by the lung tissue during ventilation which is responsible for damage. Dissipated energy is reduced even more than applied mechanical power (as currently calculate) during FCV because flow peaks are absent not only during inspiration (in contrast to PCV) but also during controlled expiration (in contrast to PCV and VCV) [32].

It may be worth noting that there was a trend towards higher SVRI, PVRI and lower CI in the FCV group (Table 3). This trend is consistent with findings in lung healthy animals [8] and lung healthy humans undergoing cardiac surgery [13] but became significant in an oleic acid ARDS model [10]. However, lactate levels and required norepinephrine doses were lower in FCV animals. The reason for this finding is not clear at the moment.

Our study has several limitations: First, results of a porcine model using insufflated air to simulate intra-abdominal hypertension cannot directly be transferred to a clinical setting. In particular, the pigs' inherently low compliance led to exceptionally high peak pressures and the aggregate viscoelastic properties of the insufflated abdomen/thoracic system are almost certainly different to those that would be found in humans in a clinical setting. Second, the observation period was relatively short. While this allowed us to investigate the short-term consequences of FCV vs PCV on respiratory mechanics and haemodynamic physiology, we were unable to estimate long-term effects such as ventilator-induced lung injury leading to respiratory failure. We showed in a previous study [10] that short time periods like this are likely too short to detect changes in inflammatory markers or cytokines, which is why we opted not to perform these measurements. Third, we chose PCV as a comparison group because it is a commonly used form of ventilation in intensive care and its gas flow profile is the exact opposite of FCV ventilation. Notwithstanding that the most novel difference between FCV ventilation and conventional methods is the control of expiration, we are not able to extrapolate our results to speculate on possible differences between FCV and VCV ventilation. Fourth, CT scans were taken during only a short inspiratory hold, where the set peak pressure was different between FCV (median 46 cmH_2_O) and PCV (median 41 cmH_2_O)—because the peak pressures used in the two methods were different owing to the individualization technique used in FCV. This may influence evaluation and interpretation of lung tissue aeration.

## Conclusion

In this porcine IAH-model we found that individualised FCV gave similar oxygenation to PCV but led to increased ventilation efficiency with a lower minute volume required to obtain normocapnia. This was accompanied by a remarkable reduction of applied mechanical power, which may be beneficial in terms of lung protection. Additionally, lung aeration was improved at IAH grade 3 with FCV compared to PCV.

## Data Availability

The dataset used and analysed during the current study are available from the corresponding author on reasonable request.

## Abbreviations

ARDS: Acute respiratory distress syndrome
BL: Baseline
CI: Cardiac index
CO: Cardiac output
CT: Computed tomography
FiO: Fraction of inspired oxygen
HR: Heart rate
HU: Hounsfield unit
IAH: Intra-abdominal hypertension
ICU: Intensive care unit
I:E: Inspiration to expiration ratio
MAP: Mean arterial pressure
MD: Median difference
MP: Mechanical power
MPAP: Mean pulmonary arterial pressure
MV: Minute volume
PCV: Pressure-controlled ventilation
PEEP: Positive end-expiratory pressure
*P*_peak_: Peak pressure
PVR(I): Pulmonary vascular resistance (index)
RR: Respiratory rate
SVR(I): Systemic vascular resistance (index)
VCV: Volume-controlled ventilation
*V*_T_: Tidal volume
